# Is the human chin a spandrel? Insights from an evolutionary analysis of ape craniomandibular form

**DOI:** 10.1371/journal.pone.0340278

**Published:** 2026-01-29

**Authors:** Noreen von Cramon-Taubadel, Jill E. Scott, Chris A. Robinson, Lauren Schroeder

**Affiliations:** 1 Buffalo Human Evolutionary Morphology Lab, Department of Anthropology, University at Buffalo, Buffalo, New York, United States of America; 2 Department of Sociology and Anthropology, Metropolitan State University of Denver, Denver, Colorado, United States of America; 3 Department of Biological Sciences, Bronx Community College, City University of New York, Bronx, New York, United States of America; 4 Doctoral Program in Anthropology and The New York Consortium in Evolutionary Primatology (NYCEP), CUNY Graduate Center, New York, New York, United States of America; 5 Department of Anthropology, University of Toronto Mississauga, Mississauga, Ontario, Canada; 6 Human Evolution Research Institute, University of Cape Town, Rondebosch, South Africa; Ohio State University, UNITED STATES OF AMERICA

## Abstract

Humans are unique among primates in possessing a chin, yet it is currently unclear whether the form of the symphyseal region of the mandible where the chin is located is the product of direct selection or a by-product of evolutionary pressures on other craniomandibular features. Here, we conduct an evolutionary analysis of hominoid craniomandibular traits to test three hypotheses: symphyseal mandibular traits evolved (1) neutrally due to genetic drift, (2) under direct selection, and (3) as a by-product (or “spandrel”) of selection on other craniomandibular traits. Evolutionary rates of morphological change, via Lande’s generalized genetic distance, were estimated along each branch of a fully-resolved hominoid phylogeny to reveal patterns of neutral, stabilizing and directional selection. Directional selection was detected along the branch between humans and the last common ancestor of chimpanzees and humans, against a backdrop of pervasive stabilizing selection and neutral evolution in hominoids. Significant directional selection was found on cranial traits reflecting increased basicranial flexion, neurocranial expansion, and reduction in lower facial prognathism, and on mandibular traits that generate a more parabolic-shaped, gracile mandible with a smaller ramus and shallower corpus. In contrast, of the nine mandibular “chin” traits, only three were under significant direct selection, while the other six were either under no selection or indirect selection. Thus, the results are consistent with the hypothesis that the symphyseal morphology that forms the human chin evolved largely as a by-product (i.e., spandrel) of direct selection for reduced anterior dental size and the craniofacial changes correlated with the evolution of bipedalism in hominins, rather than as a specific adaptation.

## Introduction

The human chin is a unique morphological feature of our species and is often used as a (if not *the*) diagnostic criterion for identifying *Homo sapiens* in the hominin fossil record [1–5]. In simplest terms, a chin is an anterior bony projection of the lower jaw (mandible) extending beyond the lower incisors [1]. However, there is surprising variability in how chin morphology is delineated [6], which has contributed to the plethora of hypotheses put forward to explain the evolution of this distinctive human morphological trait [2]. If we simply characterize the possession of a chin by the degree of protrusion of the mandibular midline (symphysis) beyond the dentition (e.g., [7]), this can result in some modern humans being deemed “chinless” if their symphyseal protrusion is relatively slight and/or their lower dentition has a relatively strong forward projection [8]. For this reason, more specific anatomical definitions of the human chin have been proposed. For example, Schwartz and Tattersall [4] argue that a true chin is an inverted T-shaped bony structure composed of a triangular protrusion at the anterior base of the mandible (*trigonum mentale*), a vertical midline keel, and two depressions (*mental fossae*) on either side of the keel. This combination of anatomical features is found only in modern humans (i.e., it is a human autapomorphy) and not in other recent hominin taxa, such as Neanderthals [4]. Others emphasize the ontogenetic formation of the human chin as being the byproduct of interaction between the forward growth of the lower (basal) part of the mandibular symphysis, and the constrained growth of the upper (alveolar) part of the anterior mandible containing the dentition [7,9,10]. Given the need to maintain occlusion between the upper and lower teeth, the alveolar region develops relatively autonomously with respect to other regions of the mandible (*sensu* [11]), and it is the higher rates of bone deposition in the basal region combined with higher rates of bone resorption in the alveolar region that result in the possession of a bony projecting chin.

Debates over how best to define chins are not trivial, as characterization of chin morphology has a direct impact on how we understand its evolutionary origins [12]. In reality, chins are complex phenotypes with an underlying polygenic and pleiotropic genetic architecture that is not well understood [13]. Thus, in order to subject chin morphology to comparative evolutionary analyses, the continuous variability of symphyseal morphology across humans and other primates must be characterized using several quantitatively measured traits. As noted by Pampush [14], if the human chin is treated as an anatomical singularity or morphological autapomorphy (sensu Schwartz & Tattersall [4]), disconnected from mandibular variation in other primate taxa, its evolutionary origins cannot be ascertained [9].

Several evolutionary hypotheses for the origins of the human chin have been proposed. It has been suggested to be adaptive for biomechanical and functional reasons [1,15,16], which implicates the action of direct selection. Specifically, the masticatory stress hypothesis states that mandibular form is designed to resist biomechanical loads during chewing [1], and studies have found that the presence of a chin results in lower symphyseal strains (e.g., [15]), although it does not counter the effects of wishboning (lateral-transverse bending) [16], which is a source of strain on the symphysis during mastication in cercopithecoids [17–19]. The more vertically oriented symphysis and reduced symphyseal tori (the “shelves” of bone on the posterior part of the symphysis) of humans imply that wishboning became a less dominant source of masticatory stress throughout hominin evolution, presumably due to changes in incision that resulted in smaller anterior dentition in hominins compared with other hominoids [12]). Other adaptive hypotheses have been proposed centered on the notion that the demands of human speech caused changes in the stresses applied to the anterior symphyseal region due to the oblique contraction of the posteriorly anchored tongue musculature, leading to the creation of the human chin through remodeling [20] or that chins are a sexually selected signal of human mate quality [21], but none of these hypotheses have received strong support for a variety of theoretical or empirical reasons [2].

Simultaneously, it has long been suggested that the human chin is a “spandrel” (*sensu* [9]) in the sense that it is the by-product of selection on other aspects of craniomandibular and/or dental morphology, such as the reduction in the size of human teeth [5], or the differential growth of the alveolar and basal portions of the mandible [22]. Alternatively, it has been suggested that human mid-facial size decreased due to a reduction in androgen levels during ontogeny [23], which may have had the co-incidental effect of exposing the chin as an anterior projection. Another proposal is that the human chin is a by-product of selection on oral and pharyngeal soft tissues in response to increased basicranial flexion due to bipedalism and a more retracted face, which would have led to a constriction in the airway had the symphysis remained posteriorly inclined [24]. Under this scenario, chin morphology results from strains placed on the midline and basal anterior symphysis due to the need to pull the posteriorly attached tongue and other soft tissues forward and away from the airways, which would have been a necessary morphological change to prevent asphyxiation, although this hypothesis is almost impossible to test with fossil taxa.

Despite the wide range of hypotheses that have been proposed to explain the origins of the human chin, they all typically assume that selection (natural or sexual) is the primary force driving evolutionary changes in the mandible, and do not test a basic null hypothesis of neutral or stochastic evolution via genetic drift. This is despite the large body of literature illustrating that at least some aspects of hominin cranial shape evolved under neutral conditions [25–29]. A recent study [12] found evidence of a rate-shift towards a faster rate of evolution in the hominin lineage relative to other apes using two quantitative measures of chin-ness (chin-angle and chin-index), suggesting that the unique symphyseal morphology seen in modern humans is the product of selection on mandibular form in hominins, but it is unclear whether this selection was direct or indirect via responses to selection on other craniomandibular regions. Moreover, while a rate shift is indicative of the action of natural selection on these two quantitative measures of symphyseal angle, the role of genetic drift in shaping variation in overall mandibular shape is not currently well understood.

**Fig 1:**
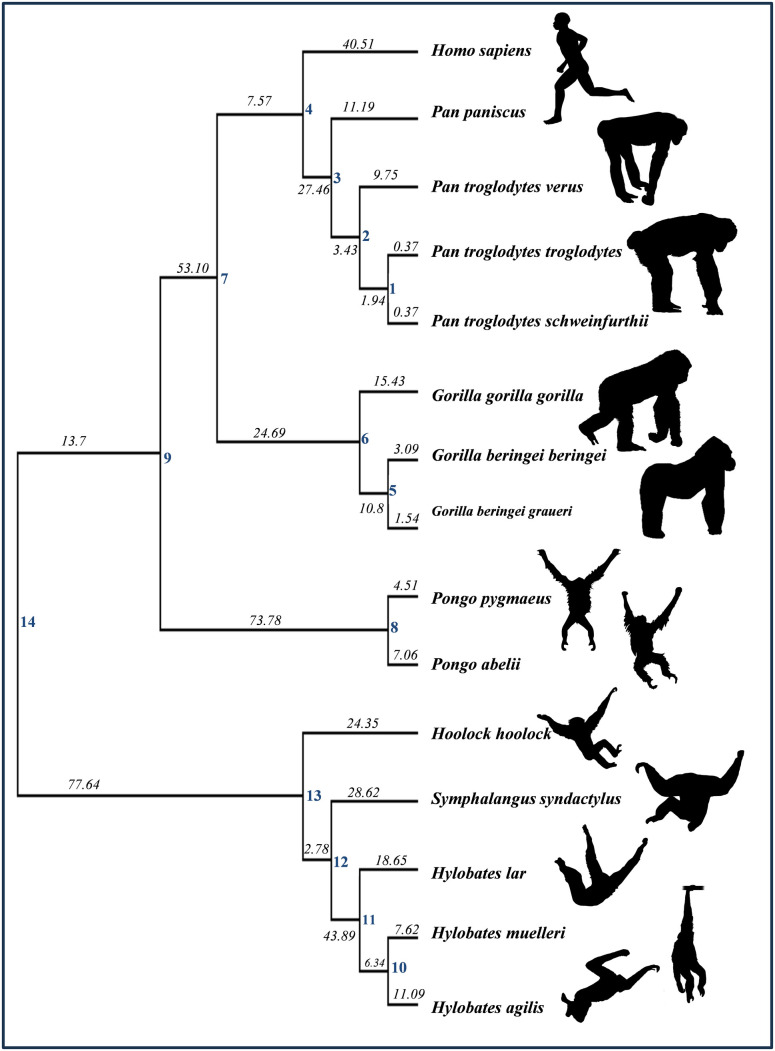
Phylogenetic tree of the fifteen hominoid taxa employed here. Maximum likelihood branch length estimates (in italics) based on Perelman et al. [35] and von Cramon-Taubadel and Smith [36]. Internal nodes (labelled 1-14) represent hypothetical ancestors.

Here we apply a quantitative genetic approach to hominoid craniomandibular traits to test the null hypothesis that the unique symphyseal morphology of the human mandible is a neutrally-evolving product of genetic drift (H_0_), with two alternative hypotheses: (H_A1_) human symphyseal morphology is formed under direct selection, or (H_A2_) human symphyseal morphology is a by-product of selection on other mandibular and/or cranial regions. It should be noted that these analyses operate within a quantitative genetics framework that assumes that all traits under consideration are subject to a polygenic and pleiotropic model of inheritance that is complex and unknown to us [30]. As such, the term “selection” is being used here to describe the non-stochastic processes of either stabilizing or directional selection acting on the statistical properties of multivariate morphological covariance matrices resulting in a degree of morphological difference between taxonomic groups that is larger or smaller than expected under a stochastic model of neutral divergence. This was achieved by calculating a phenotypic proxy for Lande’s [31] generalized genetic distance (GGD) between all branch segments of the phylogeny shown in Fig 1 following a reconstruction of mean ancestral craniomandibular trait values at each internal node [26,32–34]. Under a null hypothesis of neutrality (H_0_), Lande’s GGD follows a chi-square distribution, such that rejection at p < 0.05 signifies that morphology is evolving either slower (lower tail) or faster (upper tail) than expected, indicative of stabilizing or directional selection, respectively. If directional selection were to be detected along the branch between the last common ancestor (LCA) of humans and chimpanzees and modern humans, selection gradients and trait responses were calculated to ascertain whether traits related to symphyseal (and therefore, chin) morphology were evolving under direct (selection gradient and trait response are in the same direction; H_A1_) or indirect (selection gradient and trait response are in opposite direction; H_A2_) selection [27,28,34].

**Fig 2:**
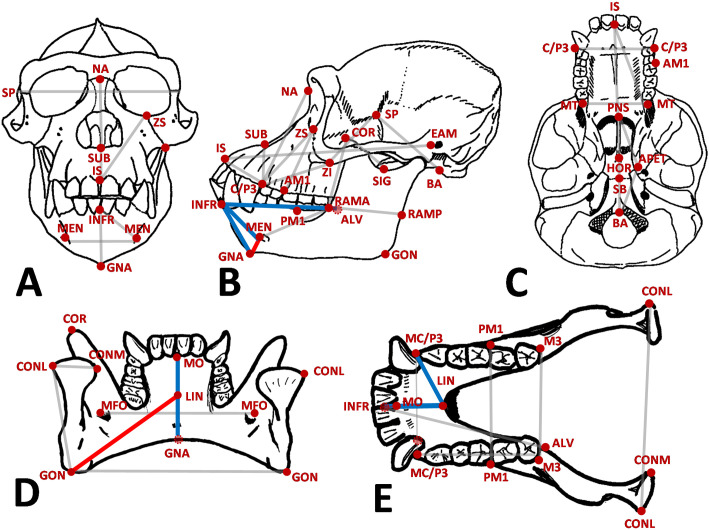
Craniomandibular landmarks with all 22 cranial and 24 mandibular interlandmark distances considered here. Landmark definitions can be found in S1 Table. Solid colored lines show the expected selection gradients to increase (red) and to decrease (blue) for the nine mandibular symphyseal traits related to the evolution of a chin under a hypothetical model of direct selection along the *Homo sapiens* branch since the last common ancestor with chimpanzees.

Under H_A1_ (direct selection) it was predicted that nine mandibular traits, directly related to symphyseal morphology, would exhibit significant selection gradients and trait responses in the directions shown in Fig 2. It should be noted that these traits encapsulate more than the specific morphology of the human chin autapomorphy and also include mandibular traits homologous across hominoids, where changes would be expected to be concentrated if the human-specific chin had evolved under direct selection. Therefore, while the human chin would be better characterized by a more localized set of traits (including for example, the human-specific anatomical landmark – pogonion), our goals are to identify whether the symphyseal morphology related to the emergence of the human chin evolved stochastically, or under direct or indirect selection, within the wider context of hominoid craniomandibular evolution. This necessitated the use of a more generalized set of homologous mandibular traits, including a sub-set that specifically relate to symphyseal morphology. Specifically, the following traits would be expected to be under direct selection to decrease: the length of the alveolar portion of the mandible (INFR-ALV), the height of the midline symphysis (INFR-GNA), the distance from the front of the anterior dentition to the mental foramen (INFR-MEN), the distance from the front of the anterior dentition to the back of the inferior transverse torus (INFR-LIN), the distance from the mental foramen to the back of the inferior transverse torus (MEN-LIN), the distance from the back of the anterior dentition to the back of the inferior transverse torus (MO-LIN), and the distance from the back of the inferior transverse torus to the bottom of the symphysis (LIN-GNA). Simultaneously, the following chin traits would be expected to be under direct selection to increase: the distance between the mental foramen and the bottom of the midline symphysis (MEN-GNA), and the internal length from the back of the inferior transverse torus to the most posterior point on the basal portion of the mandible (LIN-GON). A hypothesis of indirect selection H_A2_ would be supported if chin traits were either under no selection or under significant selection but with trait responses in opposite directions.

## Materials and methods

The dataset comprises 532 matched adult crania and mandibles of 15 hominoid taxa representing every hominoid genus besides *Nomascus* (Fig 1; S2 Table) [34,36]. No permits were required for the described study, which complied with all relevant regulations. A total of 32 landmarks (15 cranial; 17 mandibular, S2 Table) were captured directly from museum specimens using a hand-held Microscribe™ digitizer by a single observer (NvCT) and 46 (22 cranial; 24 mandibular) interlandmark distances were extracted from these landmarks (Fig 2; S1 Table). Intra-observer error was found to be less than 1 mm for all landmarks [36], which is deemed acceptable for biological landmark configurations of this size [37]. All data were logged prior to analysis to account for difference in size among hominoid species and among traits, while still preserving information about the relative form (shape and size) of the specimens [34,38]. Craniomandibular data can be found in S3 Table and hosted by Dryad.

The same fully-resolved genetic phylogeny for the 15 hominoid taxa as was used in Schroeder & von Cramon-Taubadel [34] was employed here (Fig 1). The tree is based primarily on maximum likelihood (ML) branch lengths calculated by Perelman et al. [35], supplemented with ML branch length estimates obtained from other published sources [39–42]. A complete description of how the tree was constructed can be found in von Cramon-Taubadel & Smith [36]. The pairwise ML genetic distance matrix used to construct the tree can be found in S4 Table.

A Brownian-motion maximum likelihood approach [43] was used to estimate 95% mean ancestral values for each of the 46 craniomandibular traits at each of the 14 internal nodes shown in Fig 1. This method assumes that each trait evolved under a constant stochastic Brownian motion evolutionary model, such that the sum of the squared changes along each internal branch is minimized, and evolutionary change is proportional to branch length. Schroeder & von Cramon-Taubadel [34] also tested a multiple variance Brownian motion approach to ancestral trait estimation (e.g., [44]), which allows for different rates of change across branches, and found that ancestral trait estimates did not change substantially, nor did it impact the assessment of which branches or which traits were under directional selection. Therefore, while we acknowledge that different methods of ancestral trait estimation can produce biased results depending on the assumptions of the underlying model (e.g., squared-change parsimony, Ornstein-Uhlenbeck (OU) or Brownian motion), the structure of the tree, and changes in rates of evolution in different parts of the tree [44–47], for the purposes of this study we chose to use a standard Brownian motion approach for ancestral trait estimation such that the results obtained here would be more directly comparable to those of Schroeder & von Cramon-Taubadel [34], which used a similar cranial trait dataset but did not include mandibular data. Ancestral form estimation was carried out using the *ace* function in the *ape* package in R 4.1.2 [48]. Estimated mean trait values for each ancestral node can be found in S5 Table.

The phenotypic proxy for Lande’s “generalized genetic distance” (GGD) [31] was calculated for each section of the phylogenetic tree shown in Fig 1, in order to estimate rates of morphological change among internal nodes and between shared ancestral nodes and tip taxa. Lande’s GGD is effectively a squared Mahalanobis’ distance scaled by the effective population size divided by the number of generations since divergence and can be written as:


$$\left(\frac{N_{e}}{t}\right){\left[z_{i}-z_{j}\right]}^{\prime }W^{-\text{1}}\left[z_{i}-z_{j}\right]$$

where *N*_*e*_ is effective population size, *t* is the number of generations since divergence, [*z*_*i*_ *– z*_*j*_] is the difference between trait means of ancestor/descendant pair *i* and *j*, and *W*^*-1*^ is the inverse of the pooled within-group phenotypic variance-covariance (V/CV) matrix (P) substituting for the additive genetic covariance matrix G [49]. The practice of substituting the P-matrix for the G-matrix in cases where estimates of genetic covariance are unattainable, known as Cheverud’s conjecture, has generally been found to be valid for morphological traits as long as the average heritability is reasonably high and the sample size used to construct the P-matrix is ≥ 40 [50]. Pooled within-group phenotypic V/CV matrices (*W*) were calculated using all extant taxa involved in each ancestral trait estimation. This approach is justified based on previous studies that have found highly conserved patterns of cranial trait covariance across hominoids [51,52] and indeed mammals on the whole [53]. For example, *W* for the GGD calculation between node 9 (*Pongo* ancestor) in Fig 1 and tip taxon *Pongo pygmaeus* was based on the pooled within-group V/CV using all *Pongo* specimens. The minimum sample size used to construct any phenotypic V/CV matrix was 61 (combination of two *Pongo* species), which is above the sample size threshold generally needed to generate stable P-matrix estimates [54]. The potentially confounding effects of sex and taxonomic structure on *W* were corrected for using the residuals from a MANOVA with sex and taxon as independent variables. In addition, *W* was multiplied by 0.4 to take account of the incomplete heritability of primate cranial traits [34,55]. The value of 0.4 was taken as an average measure of primate cranial trait heritability based on the limited number of relevant studies conducted [49,55–59]. Assuming incomplete heritability is conservative in this analysis, as assuming full heritability would risk overestimating the rate of evolution (i.e., generate larger GGD estimates). GGD follows a chi-square distribution under a model of stochastic genetic drift, with degrees of freedom equivalent to the number of traits [31]. Small values that reject a null hypothesis of drift indicate the effects of stabilizing selection as the amount of morphological divergence is less than expected under drift, while large significant values indicate the action of directional selection (i.e., more morphological change than expected). Here rates were designated as “very slow”, “very fast”, “slow” and “fast” in a two-tailed test at 99.9% and 95% confidence intervals, respectively.

Estimates of effective population size (*N*_*e*_) and number of generations since divergence (*t*) are the same as those used by Schroeder & von Cramon-Taubadel [34] for each ancestral node in order to provide comparable results with the latter study. *t* is calculated as time since divergence corrected for average generation length. It should be noted that a recent study based on whole genome data [60] provides updated estimates of divergence times for some of the same nodes considered here. The means and ranges from this recent study are included in S6 Table, which provides all the parameter values used and a description of where the data were collated from. It is worth noting that except for the divergence of the two orangutan (*Pongo*) species, all of our assumed divergence times fall within the confidence intervals estimated by Shao et al. [60], providing further support that these divergence estimates are relatively robust. Even in the case of *Pongo*, our divergence time estimate (1.3 mya) falls just outside the lower bound of the range (1.52–3.88 mya) provided by Shao et al. [60], which is considerably older than other published divergence times (e.g., 0.97 mya) for orangutan species [61]. As in Schroeder & von Cramon-Taubadel [34], we calculated 95% confidence intervals for each estimate of *N*_*e*_, and GGD distances were also estimated for these confidence intervals to ensure that GGD values were not biased by any errors in the calculation of effective population size.

In instances where GGD values indicated fast rates of morphological evolution, and neutral divergence could be rejected (i.e., “fast” or “very fast” GGD values), the magnitude and pattern of selection required to produce the differences between estimated ancestral and descendant taxon means was reconstructed. The differential selection gradient [31,62] was estimated using the following equation:


$$\beta =\;W^{-\text{1}}\left[z_{i}-z_{j}\right]$$

Where β is the multivariate selection gradient, $W^{-\text{1}}$ is the inverse pooled within-group phenotypic V/CV matrix, and $\left[z_{i}-z_{j}\right]$ is the vector of mean differences between ancestral and descendent taxa *i* and *j* [28,34,51]. 95% confidence intervals were calculated using a resampling approach to calculate 1000 vectors of trait means that were then used to estimate a distribution of 1000 selection gradients (β) with the differential selection gradient equation above. Selection gradients were considered statistically significant if their 95% confidence intervals did not overlap with zero. Overlapping with zero would indicate that the estimated selection gradient was negligible and therefore non-significant. These selection gradients were used to differentiate between direct and indirect selection, as the selection gradient is a vector of partial regression coefficients of relative fitness onto the traits [63]. Positive selection gradients signify a positive relationship with fitness if the trait response is also positive (i.e., selection is favoring the trait to increase in size and the trait is responding appropriately). Negative selection gradients signify a negative proportional relationship with fitness if the trait is also negative (i.e., selection is favoring the trait to decrease in size and the trait is responding appropriately). Both of these indicate direct selection with a matched trait response. However, if the trait response is in the opposite direction of the gradient, then it indicates that the trait has no relationship with fitness and is not under direct selection. Instead, it is likely that the change in the trait mean is uncorrelated with the selection gradient and instead is responding to selection on other traits with which is covaries.

## Results

### Rates of morphological evolution across apes

Rates of evolution indicate that, for the majority of branches across the hominoid phylogeny, craniomandibular morphological divergence was slow and departures from the neutral model occurred mainly in the form of stabilizing selection indicated by green GGD values in Table 1. This is comparable to what was found by Schroeder & von Cramon-Taubadel [34] for hominoid cranial form. There were a few instances where genetic drift could not be rejected (marked in yellow in Table 1), most notably between node 3 (*Pan* ancestor) and *Pan paniscus*, and between node 8 (*Pongo* ancestor) and *Pongo abelii.* Fast rates of morphological change, indicative of directional selection (red GGD values in Table 1), were detected in the branch between node 4 (LCA of *Homo* and *Pan*) and *Homo sapiens.* While not as strong, there was also some evidence of directional selection in the divergence of the great apes from their last common ancestor with the lesser apes (between nodes 13 and 14), and the divergence of mountain gorillas (*Gorilla b. beringei*) from the other eastern gorilla subspecies. However, unlike the results for humans, these latter two cases of directional selection were only found for GGD values based on mean and upper bound estimates of effective population size scaled by number of generations since divergence (N_e_/t), suggestive of the influence of changing effective population sizes in the evolution of these lineages [34].

**Table 1:** Generalized genetic distances (GGD) along each branch of the phylogenetic tree shown in Fig 1.

Node	Node/*Taxon*	Morph Diff.	N_e_/t mean	GGD	N_e_/t lower	GGD	N_e_/t upper	GGD
**1**	*P. t. troglodytes*	14.47	1.11	16.06	0.61	8.83	1.62	23.44
**1**	*P. t. schweinfurthii*	13.88	1.11	15.40	0.61	8.47	1.62	22.48
**2**	*P. t. verus*	46.77	0.37	17.31	0.23	10.76	0.50	23.39
**2**	**1**	08.73	0.37	3.23	0.23	2.01	0.50	4.36
**3**	*Pan paniscus*	150.91	0.27	***40.75***	0.18	27.16	0.35	***52.82***
**3**	**2**	13.63	0.27	3.68	0.18	2.45	0.35	4.77
**4**	*Homo sapiens*	669.03	0.18	**120.43**	0.14	**93.66**	0.23	**153.88**
**4**	**3**	173.06	0.18	31.15	0.14	24.23	0.21	***36.34***
**5**	*Gorilla b. beringei*	113.07	0.69	**78.02**	0.40	***45.23***	0.99	**111.94**
**5**	*Gorilla b. graueri*	23.57	0.69	16.26	0.40	9.43	0.99	23.33
**6**	*Gorilla gorilla*	74.78	0.26	19.44	0.18	13.46	0.33	24.68
**6**	**5**	43.71	0.26	11.37	0.18	7.87	0.33	14.43
**7**	**6**	164.44	0.15	24.67	0.12	19.73	0.18	29.60
**7**	**4**	12.92	0.15	1.94	0.12	1.55	0.18	2.33
**8**	*Pongo pygmaeus*	49.88	0.36	17.96	0.23	11.47	0.50	24.94
**8**	*Pongo abelii*	134.15	0.36	***48.29***	0.23	30.85	0.50	**67.08**
**9**	**7**	89.87	0.15	13.48	0.12	10.78	0.18	16.18
**9**	**8**	208.54	0.15	31.28	0.12	25.02	0.18	***37.54***
**10**	*Hylobates muelleri*	38.09	0.16	6.09	0.13	4.95	0.19	7.24
**10**	*Hylobates agilis*	35.67	0.16	5.71	0.13	4.64	0.19	6.78
**11**	*Hylobates lar*	34.13	0.14	4.78	0.12	4.10	0.17	5.80
**11**	**10**	6.18	0.14	0.87	0.12	0.74	0.17	1.05
**12**	*Sym. syndactylus*	65.41	0.23	15.04	0.16	10.47	0.29	18.97
**12**	**11**	113.92	0.23	26.20	0.16	18.23	0.29	***33.04***
**13**	*Hool. hoolock*	46.40	0.18	8.35	0.14	6.50	0.23	10.67
**13**	**12**	0.43	0.18	0.08	0.14	0.06	0.23	0.10
**14**	**9**	15.52	0.14	2.17	0.12	1.86	0.16	2.48
**14**	**13**	498.49	0.14	**69.79**	0.12	***59.82***	0.16	**79.76**

Colors correspond to neutral (yellow, bold italics), fast/very fast (pink/red, bold) and slow/very slow (light/dark green, plain) evolutionary rates. Nodes are labelled as in Fig 1. Morphological differences (Morph Diff.) refer to Mahalanobis’ distances between pairs of nodes, or nodes and tip taxa. GGD scales Mahalanobis’ distances by effective population size (N_e_) as a ratio of number of generations since divergence (t). Primary GGD results were generated for estimated mean N_e_/t as well as the upper and lower bounds of the 95% confidence intervals for the estimation of N_e_ given in S6 Table.

### Assessing direct and indirect selection in the human cranium

**Fig 3:**
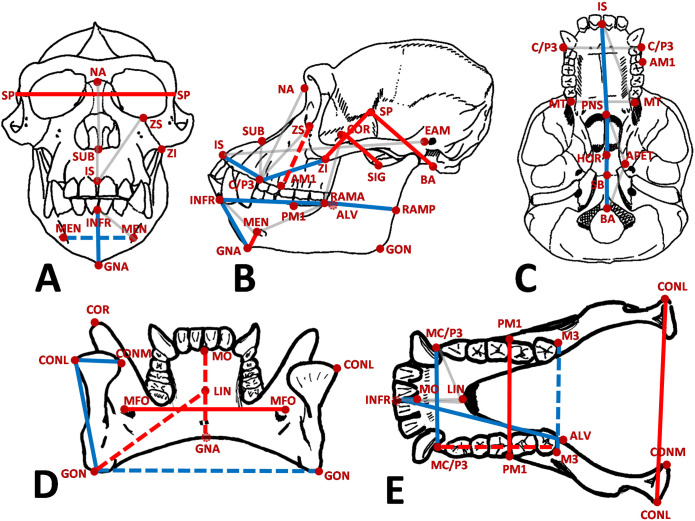
Craniomandibular traits found to be under significant direct selection (solid lines) to increase (red) and decrease (blue). Traits marked in either red or blue dashed lines were found to be under indirect selection. Traits in light grey did not return significant selection coefficients (Table 2).

Given that the null hypothesis (H_0_) of neutral morphological divergence under genetic drift could be rejected for the human cranium and mandible (and by extension the chin), the two alternative hypotheses (H_A1_ and H_A2_) of direct and indirect selection were tested. In the cranium (Table 2, Fig 3), significant direct selection for trait size increase was found in traits related to basicranial flexion (e.g., lengthening of SP-BA, SP-ZI) and expansion of the neurocranium (SP-SP), while significant direct selection for trait size decrease was primarily related to facial retraction (IS-C/P3, ZI-C/P3, IS-PNS), and the anterior migration of the foramen magnum and increased basicranial flexion (SB-BA, HOR-SB, PNS-HOR) associated with the shift towards a bipedal mode of locomotion. Only one trait (ZS-AM1) was under indirect selection whereby the height of the midface was under selection to increase but the trait actually decreases. This makes sense as the trait also reflects the angle of the face (the position of the first molar relative to the base of the orbit), which is more orthognathic in humans compared with other great apes, yet the absolute size of the face also decreases in the human lineage resulting in an absolutely smaller trait value even if the trait is under selection to increase.

**Table 2:** Selection gradients (β) and responses (% change in trait means) for all traits from the LCA of *Pan*-*Homo* to humans arranged in descending order of β.

Trait	Selection Response (% ± in trait mean)	Selection gradient (β)*	Selection gradient (95% Confidence Intervals)	
**CRANIAL**				
SP-BA	3.8541	515.4307*	585.6914	435.4855
SP-SP	7.3461	287.7135*	368.9685	208.8200
*ZS-AM1*	−5.8710	241.7930*	369.0288	122.3056
IS-MT	−5.3775	209.0735	415.2773	−13.8035
SP-ZI	2.3945	138.4690*	206.8997	73.1346
IS-ZS	−6.1206	138.0642	364.2501	−71.4618
PNS-APET	−2.3420	65.2389	171.7813	−12.7931
APET-BA	−4.4711	42.9439	127.6374	−38.7342
SUB-C/P3	−6.8819	26.5880	200.3735	−146.5171
MT-MT	2.4157	−11.1437	43.6214	−77.1778
NA-C/P3	−3.6591	−37.7435	458.7662	−390.2743
SUB-IS	−12.3696	−41.0535	33.7996	−131.8543
HOR-SB	−17.5308	−49.1155*	−15.9273	−86.6819
C/P3-C/P3	−5.9169	−85.3321	51.3500	−219.9699
ZI-C/P3	−3.5872	−98.8894*	−15.4432	−178.5268
NA-SUB	−3.8179	−111.7796	129.6796	−437.9244
ZS-C/P3	−5.9840	−158.0656	54.0436	−398.4203
SB-BA	−2.8132	−163.8733*	−97.9905	−233.0557
PNS-HOR	−3.8184	−190.4819*	−114.2027	−268.5657
IS-EAM	−4.1750	−217.8403	62.4940	−537.0252
IS-C/P3	−9.8146	−235.5367*	−45.9959	−414.2599
IS-PNS	−7.1524	−353.8053*	−177.7926	−536.5646
**MANDIBULAR**				
PM1-PM1	2.2879	526.1893*	642.75432	389.56733
*MC/P3-M3*	−0.4185	301.3210*	389.07869	197.13451
MFO-MFO	1.7690	211.1005*	305.90163	129.04577
***LIN-GON***	−3.8791	183.9250*	305.67458	59.61838
**MEN-GNA**	1.7183	172.9049*	250.18564	106.85773
CONL-CONL	0.5172	164.5703*	251.04382	76.82849
***MO-LIN***	−5.8825	131.0050*	202.14646	67.67265
***LIN-GNA***	−10.1542	66.1226*	109.57331	19.77782
**INFR-MEN**	−5.9248	46.8370	195.08516	−100.59222
**INFR-LIN**	−7.3079	39.6416	144.68381	−47.81651
MEN-RAMA	−2.9555	38.9883	103.28186	−23.33838
COR-SIG	2.6945	38.4653*	68.61851	11.24071
**MEN-LIN**	−1.1773	24.9773	169.71859	−126.73927
COR-RAMA	−7.8700	−9.7928	32.36041	−57.81069
INFR-MO	−14.0403	−44.4173*	−17.73015	−83.96758
CONL-GON	−4.3920	−75.4156*	−11.60225	−131.89832
*GON-GON*	1.0090	−100.3395*	−59.36917	−137.77858
CONL-CONM	−5.6126	−111.2406*	−63.46066	−155.72495
RAMA-RAMP	−7.3824	−116.7033*	−38.05380	−211.12342
*M3-M3*	3.3227	−155.2085*	−20.75275	−282.38384
MC/P3-MC/P3	−5.7914	−156.7796*	−64.34690	−285.82365
*MEN-MEN*	1.1017	−204.3162*	−64.24228	−349.76302
**INFR-GNA**	−13.2318	−244.1441*	−183.21974	−325.88167
**INFR-ALV**	−4.2994	−339.5208*	−197.73120	−459.30422

*Significant selection gradients (i.e., where the 95% confidence intervals do not overlap with zero). Red shading = selection for trait to increase; blue shading = selection for trait to decrease; italics = selection gradient and responses are opposite in direction, indicative of indirect selection. All % selection responses > ±1σ from the mean are underlined. Mandibular traits specifically related to symphyseal morphology are bolded.

### Assessing direct and indirect selection in the human mandible

More mandibular traits (19 out of 24) were found to have significant selection gradients compared with only ten out of 22 cranial traits (Table 2). In the mandible, there was significant direct selection for size increase on traits related to the widening of the posterior aspect of the mandible (CONL-CONL, MFO-MFO, PM1-PM1) and significant direct selection for trait size decrease on transverse dimensions of the anterior mandible (MC/P3-MC/P3), which together change a U-shaped ape-like mandible to a more parabolic human mandible (Table 2, Fig 3). There was also significant direct selection for decrease on the height (CONL-GON) and width (RAMA-RAMP) of the ramus, and on the width of the mandibular condyle (CONL-CONM), relating to the overall reduction in the size of, and gracilization of, the human mandible [64]. Direct selection for trait size decrease was also detected around the anterior dentition (INFR-MO, MC/P3-M/CP3). Direct selection was found to increase the height of the coronoid process (COR-SIG) commensurate with the relatively longer coronoid process and deeper sigmoid notch found in humans relative to *Pan* [65]. Several non-symphyseal traits were found to be under indirect selection: the posterior transverse width of the basal portion of the ramus (GON-GON) and the transverse width at the third molar (M3-M3) were under selection to decrease but actually increased in size, a response that corresponds with the more parabolic shape of the human mandible. There is an overall reduction in the size of the human mandible relative to the estimated last common ancestor resulting in the vast majority of trait responses being negative, so these positive trait responses are noteworthy. Finally, the length of the posterior tooth row (MC/P3-M3) was found to be under selection to increase, yet the trait response was negative. The positive selection gradient is most likely related to the relative increase in the size of the posterior teeth compared with the anterior teeth in hominins compared with other ape taxa [66], yet the overall response is a reduction in mandibular (and dental size) such that the response does not match the selection gradient. Indeed, this exemplifies how indirect selection can have an effect on traits. Although a trait may have experienced a strong positive selection gradient, a negative response is possible due to trait integration with other traits (as captured by the covariance structure among traits). In such a case, the negative response may result from a strong negative selection gradient acting on other correlated traits (presumably with negative responses) which in turn constrains the response through their shared covariance. Nevertheless, overall, our results for the mandible align with previous studies that found evidence of rapid evolution in mandibular shape in the human lineage relative to other hominoid taxa [14,66].

In terms of the nine mandibular traits directly associated with symphyseal morphology that are related to the evolution of the chin (Table 2), only three traits (MEN-GNA, INFR-GNA and INFR-ALV) followed the predictions made under a hypothesis of direct selection (Fig 2). Three traits (INFR-MEN, INFR-LIN and MEN-LIN) were not found to be under any significant selection, while the remaining three traits (LIN-GNA, LIN-GON, MO-LIN) were found to be under indirect selection.

## Discussion

### Is the chin a spandrel?

Taken together, the results are not fully consistent with the hypothesis that the human chin is the product of direct selection (H_A1_) but rather align more closely with the second alternative hypothesis (H_A2_) of indirect selection. This conclusion is based on the fact that only three out of nine chin-related symphyseal traits were found to be under direct selection, while the remaining six traits were either under no selection or indirect selection. This implies that most human symphyseal traits have evolved largely as an evolutionary side-product or “spandrel” due to covariation with other mandibular and cranial traits that are under direct selection, including the three “chin” traits found to be under direct selection (i.e., height of the symphysis, length of the alveolar region, and the location of the base of the symphysis relative to the position of the mental foramen).

### The pattern of direct selection on craniomandibular form aligns with expectations

If we look at the overall pattern of directional selection in the cranium and mandible (solid blue and red traits in Fig 3), some clear morphological patterns emerge. Selection has acted to increase basicranial flexion, increase the width of the lateral neurocranium (with an associated increase in the width of the posterior mandible), decrease the size of the lower face (especially around the anterior dentition), decrease the length of the alveolar portion of the mandibular corpus, reduce the overall height of the mandibular corpus, and reduce the height and width of the mandibular ramus. These are all familiar morphological changes associated with the evolution of bipedalism and the reduction in the size of the anterior dentition in early hominins compared with the last common ancestor of chimpanzees and humans [67].

While our results illustrate which aspects of craniomandibular form have been under the most potent direct selection in the hominin lineage since the last common ancestor with chimpanzees, the relative timing of these changes remains unclear. Some of the major changes identified above (basicranial flexion and dental/facial reduction) occur, at least initially, in early hominin evolution, yet the distinctive chin is found only later in modern humans. This implies that some of the direct selection pressures identified here persisted for most (if not all) of hominin evolution (see also [66]), while others may have occurred at different points in time. For example, the general pressure to decrease the size of, and the projection of, the lower face may have been a long-term one, first in response to the reduction in the size of hominin incisors and canines relative to the *Pan-Homo* ancestor [68], and then later a general reduction in the size of the dentition in response to a shift towards a less mechanically-challenging diet including more meat and/or cooked foods [8,69–73]. This long-term selection pressure on the relative size of the lower face and dentition would eventually have contributed to the evolution of the human chin via a pattern of mandibular bone growth whereby bone deposition occurred on the anterior side of the basal portion of the corpus, potentially to help maintain structural integrity while under strains from chewing, while the alveolar region experienced slower rates of bone deposition and/or bone resorption, especially in the anterior symphyseal region [5,9,22]. This scenario is consistent with the results of Pampush and colleagues [12] who found a significantly higher rate of symphyseal angle evolution early in the hominin lineage during the evolution of the australopithecines. The symphyseal angle is the angle between the alveolar plane (INFR-ALV) and a line drawn between the landmarks infradentale and gnathion (INFR-GNA). Their results suggest that the shift from a receding ape-like symphysis (more acute angle) to a more upright hominin-like symphysis (more obtuse angle) began early in human evolution, with the more extreme basal projection seen in modern humans being an exaggeration of a general hominin evolutionary trend [12]. It is interesting to note that while we did not use the same measure of symphyseal angle here, the symphyseal angle can be calculated from the relative position of two of the three “chin” traits that we found to be under direct selection. Essentially, all else being equal, if the anterior-posterior length of the alveolar region (INFR-ALV) is under strong selection to decrease in length (due, for example, to selection to decrease tooth size) but the relative position of gnathion (base of the symphysis) is under constraint to maintain its anterior position for biomechanical reasons, the symphyseal angle will become more upright as a consequence. In that sense, our results are in agreement with those reported by Pampush et al. [12]. While this shift in symphyseal angle alone does not create the unique human chin, it does pave the way for the later development of the human chin as other craniomandibular changes occur. However, identifying when and in which taxa correlated morphological changes in the cranium and mandible occurred requires incorporating fossil hominin craniomandibular evidence into an analytical framework such as the one presented here.

### Morphological integration is key to understanding the evolution of craniomandibular form

When viewed through a multivariate quantitative genetic lens (i.e., the statistical analysis of quantitative trait covariation), many of the seemingly competing hypotheses for the origins of the human chin may, in fact, be simultaneously correct. Defining the human chin as a singular “trait” (e.g., an inverted T-shaped protrusion, [4]), that is deemed to be either present or absent, places a severe limitation on the ability to track its evolutionary history [6,13], especially as it is considered to be an autapomorphy of *Homo sapiens*. So, while narrow definitions of what constitutes a chin might aid in deciding whether a particular hominin fossil counts as being “modern human” or not, it does not address the question of how or why the human chin evolved. Instead, if the human chin is viewed as the morphological consequence of a series of coordinated changes in the mandible and maxilla, and is quantified using a series of variables, it is possible to begin to tease apart which aspects of morphology have been subject to selection and which ones changed over time as a consequence of their covariation with other traits.

Overall patterns of covariation among traits are often couched in terms of the related concepts of *integration* and *modularity* (e.g., [74]), whereby integration refers to the degree and nature of covariation among particular traits [75], while modularity refers to the existence of semi-autonomous trait-complexes that exhibit tighter morphological integration due to relatively stronger patterns of pleiotropy within modules than between modules [76]. Therefore, morphological modules can emerge as quasi-independent units of evolution due to having relatively stronger genetic correlation (i.e., more pervasive pleiotropy) within them, and thus having relatively stronger patterns of trait correlation or covariance than between modules (e.g., [74,76–78].

Despite the large body of literature assessing and comparing patterns of integration and modularity across the primate cranium [30,79], relatively few studies have assessed patterns of integration and modularity in the primate mandible. Polanski [80] assessed ontogenetic changes in the patterns and degree of integration within the human mandible, and found a trend towards increased modularity of the alveolar region relative to the rest of the corpus and ramus, which remained relatively tightly integrated throughout ontogeny. In the 1960s Melvin Moss and colleagues (e.g., [11,81]) suggested that the human mandible is comprised of several distinct semi-autonomous modules, including the alveolar, basal, angular, coronoid and condyloid regions. Of these, the alveolar region is closely associated with the development of the dentition, while the basal and condyloid regions (which together make up the posterior part of the ramus and the inferior part of the corpus) have the largest independence to grow and expand the dimensions of the mandible [16]. Polanski’s [80] results concur with this and are also in agreement with previous assessments of growth and modularity in African apes [82], whereby a separation of the alveolar and ramus regions follows the basic mammalian pattern of mandibular growth [83].

Given the need to maintain proper occlusion of the maxillary and mandibular dentition [10,84], it makes sense that the mandible and the maxillary portion of the cranium should be somewhat integrated [85,86] across primates. While this does seem to be the case across anthropoid taxa, Jung & von Cramon-Taubadel [87] found that the human cranium and mandible are particularly strongly integrated compared with other taxa. There is also evidence that changes in the morphology of the temporal fossa (lateral neurocranium) are related to changes in post-canine alveolar length and mandibular robusticity in both humans and chimpanzees [88]. In the case of humans, this suggests a link between neurocranial expansion in later hominins and changes in the relative size of the alveolar region of the mandible. However, the question remains whether the correlations observed between neurocranial and maxillary morphology, and mandibular morphology are causally related or not. Polanski [80] suggests that it is the evolution of the cranium that is the driving force in generating changes in the mandible (see also [88,89]). However, our results illustrate coordinated direct selection in both the cranium *and* mandible, especially in relation to the relative length of the maxilla and alveolar portion of the mandible (Fig 3), which suggests that selection may have acted on both components simultaneously. On the other hand, if selection were acting directly on the size of both the upper and lower dentition, it would have the effect of appearing as if selection were acting on the maxilla and mandible simultaneously. Further analyses with specific basicranial traits that link the cranium with the posterior mandible would be required to address the question of whether the association between basicranial width and the creation of a parabolic-shaped mandible is being driven by selection for brain expansion in later hominins.

Therefore, while our results do not provide evidence that the human chin evolved “for” any specific purpose (such as resisting biomechanical stress during mastication, the muscular demands of human speech, or as a sexually-selected signal), it is a true “spandrel” [9] in the sense that it has adaptive value in providing sufficient biomechanical support in the basal region even as the alveolar region has continued to decrease in post-agricultural and post-industrial modern human populations [90,91]. The role of tongue musculature related to language [20] should not be discounted as it may have provided an additional selective force shaping the internal morphology of the anterior mandible given that early modern human fossils have pronounced chins but often have larger and more robust mandibles/teeth than more recent (especially post-agricultural) populations. So, while dental reduction was the most likely selection pressure acting to reorient the symphyseal angle that would eventually lead to the emergence of a true chin, it is also possible that the evolution of language in the modern human lineage accelerated the differential growth of the basilar and alveolar portions of the mandibular corpus, leading to the emergence of the unique human chin.

## Conclusions

Our results suggest that human symphyseal morphology that enables the possession of a chin is an evolutionary side-product or “spandrel” (*sensu* Gould & Lewontin [9]) resulting from direct selection on other craniomandibular regions, including increased basicranial flexion, facial reduction, neurocranial expansion, and a more gracile mandible with a more upright symphysis. These are familiar craniomandibular changes associated mostly with the early hominin fossil record, which documents the evolution of a lineage of bipedal apes with decreased anterior dentition relative to other great apes, and a concomitant reduction in the size of the maxilla and alveolar region of the mandible (e.g., [92]). However, the chin is not a singularity but rather the result of multiple changes in the relative position of different aspects of mandibular morphology. So, while a more vertical symphyseal angle associated with smaller anterior dentition was an important evolutionary change within hominins that allowed for the human chin to eventually evolve, selection on other craniomandibular regions also contributed to the emergence of this unique human trait. This underscores the importance of assessing the evolution of morphology with trait integration in mind.

## Supporting information

S1 Table List of taxa used in this study, including repository information and sample sizes. (PDF)

S2 Table Craniomandibular landmark codes and anatomical descriptions. (PDF)

S3 Table Logged cranial and mandibular data. Taxon names match those in S1 Table. Trait names match landmark names found in S2 Table and Fig 2.
(XLSX)

S4 Table Pairwise among-taxon maximum-likelihood genetic distance matrix used to construct phylogenetic tree. (PDF)

S5 Table Estimated craniomandibular trait values (with 95% confidence intervals) at each ancestral node. Trait names match those used in S3 Table. Ancestral node numbers match those used in Fig 1.
(XLSX)

S6 Table Divergence times, generation times, and effective population sizes (with 90% confidence intervals) for each ancestral node. Node numbers and ancestor taxon codes match those used in S5 Table.
(PDF)
