# Supplementary material for: Is the human chin a spandrel? Insights from an evolutionary analysis of ape craniomandibular form
Source: PLoS One. 2026 Jan 29;21(1):e0340278. doi: 10.1371/journal.pone.0340278 (PMC12854472; doi:10.1371/journal.pone.0340278)
Supplement: S1 Table — (PDF) [file pone.0340278.s001.pdf]

S1 Table. List of taxa used in this study, including repository information and sample sizes

Taxon	Repository †	Sample sizes		
		Males	Females	Total
Homo sapiens	NHM, NHMW, DC, MH, AMNH	25	25	50
Pan troglodytes troglodytes	PC	20	20	40
Pan troglodytes schweinfurthii	RMCA	20	20	40
Pan troglodytes verus	NHM, RMCA, AMNH	10	5	15
Pan paniscus	RMCA	18	22	40
Gorilla gorilla	PC	23	21	44
Gorilla beringei beringei	RMCA, AMNH, MCZ, SNMNH	14	10	24
Gorilla beringei graueri	RMCA	19	20	39
Pongo abelii	NHM, MCZ, SNMNH, BSC	12	11	23
Pongo pygmaeus	BSC	18	20	38
Hylobates lar	NHM, SNMNH	18	17	35
Hylobates muelleri	SNMNH	14	16	30
Hylobates agilis	AMNH, SNMNH	20	17	37
Symphalangus syndactylus	NHM, BSC, AMNH, SNMNH	20	19	39
Hoolock hoolock	AMNH	19	19	38

† NHM = Natural History Museum (London), NHMW = Natural History Museum (Vienna), DC = Duckworth Collection (Cambridge), MH = Musée de l'Homme (Paris), AMNH = American Museum of Natural History (New York), PC = Powell-Cotton Collection (Kent), MCZ = Museum of Comparative Zoology (Harvard), RMCA = Royal Museum of Central Africa (Tervuren), SNMNH = Smithsonian National Museum of Natural History (Washington D.C), BSC = Bavarian State Collection for Anthropology and Paleoanatomy (Munich).
