# Supplementary material for: Is the human chin a spandrel? Insights from an evolutionary analysis of ape craniomandibular form
Source: PLoS One. 2026 Jan 29;21(1):e0340278. doi: 10.1371/journal.pone.0340278 (PMC12854472; doi:10.1371/journal.pone.0340278)
Supplement: S2 Table — (PDF) [file pone.0340278.s002.pdf]

S2 Table. Craniomandibular landmark codes and anatomical descriptions

Code	Landmark Name[1]	Anatomical Description
Cranial Midline		
BA	Basion	The point where the anterior margin of the foramen magnum intersects the midsagittal plane
HOR	Hormion	The midline point of attachment of the vomer and sphenoid bones
IS	Intradentale superior (Prosthion)	The most anterior midline point on the maxillary alveolus between the two central incisors
PNS	Posterior nasal spine (Alveolon)	The point on the interpalatal suture in line with the posterior margins of the alveolar process
NA	Nasion	The point of intersection of the nasofrontal suture and the midsagittal plane
SB	Sphenobasion	The midline point on the sphenooccipital suture
SUB	Subspinale	The most inferior midline point where the nasal spine intersects with anterior edge of the maxilla
Cranial Lateral		
APET	Anterior petrous temporal (Petrosal)	The most anterior point of the petrous element of the temporal bone
AM1	Anterior M1 (Alveolare)	The most anterior point on the alveolus of the first molar
C/P3	C/P3	The most inferior point on the external surface of the alveolar margin between the canine and P3
EAM	External auditory meatus (ant)	The most anterior point on the margin of the external auditory meatus
MT	Maxillary tuberosity	The most posterior, inferior point on the maxilla
SP	Sphenion[2]	The most anterior extent of the sphenoparietal suture (pterion)
ZI	Zygomaxillare inferior (Zygomaxillare)	The most inferior, anterior point on the zygomaticomaxillary suture
ZS	Zygomaxillare superior (Zygoorbitale)	The point where the zygomaticomaxillary suture intersects with the inferior orbital margin
Mandibular Midline		
GNA	Gnathion	The most inferior midline point on the mandibular symphysis
INFR	Infradentale	The most superior midline point on the buccal surface of the alveolus
LIN	Linguale	The most superior-posterior point on the lingual superior transverse torus
MO	Mandibular orale	The most superior midline point on the lingual surface of the alveolus
Mandibular Lateral		
ALV	Alveolus (pos)	The most superior, posterior point on the alveolus
CONL	Condylion (lateral)	The most lateral point on the superior surface of the mandibular condyle
CONM	Condylion (medial)	The most medial point on the superior surface of the mandibular condyle
COR	Coronion	The most superior point on the coronoid process
GON	Gonion	The point of maximum curvature where the posterior ramus and the basal corpus intersect
MC/P3	(Mandibular) C/P3	The most lateral point on the alveolus between the canine and P3
MEN	Mental foramen (ant)	The most anterior point on the lateral edge of the mental foramen
MFO	Mandibular foramen	The most anterior, inferior point on the medial edge of the mandibular foramen

M3	M3 (lat-pos)	The most lateral point on the alveolus posterior to M3
PM1	(Posterior) M1	The most superior, posterior point on the alveolus
RAMA	Ramus (ant)	The most anterior point on the ascending ramus in line with the alveolus
RAMP	Ramus (pos)	The most posterior point on the ascending ramus in line with the alveolus
SIG	Sigmoid notch	The most superior point of maximum inflection in the depth of the sigmoid notch

All landmarks were collected by NvCT on original material.

[1]Alternative landmark names used by von Cramon-Taubadel & Smith 2012 are given in parentheses, where they differ from those used by Schroeder & von Cramon-Taubadel 2017. Anatomical descriptions follow from those provided by von Cramon-Taubadel & Smith 2012.

[2]Given the substantial sutural variation possible at Pterion in catarrhines we follow von Cramon-Taubadel & Smith 2012 in using the most anterior points (Sphenion) of Pterion irrespective of whether the sutural pattern is spheno-parietal or temporo-frontal.
