# Supplementary material for: Is the human chin a spandrel? Insights from an evolutionary analysis of ape craniomandibular form
Source: PLoS One. 2026 Jan 29;21(1):e0340278. doi: 10.1371/journal.pone.0340278 (PMC12854472; doi:10.1371/journal.pone.0340278)
Supplement: S4 Table — (PDF) [file pone.0340278.s004.pdf]

S4 Table. Pairwise among-taxon maximum-likelihood genetic distance matrix used to construct phylogenetic tree. See von Cramon-Taubadel & Smith 2012 for full details of how genetic distances were calculated.

Taxon*	Hool	GorBB	GorBG	GorGG	Homo	HyloA	HyloL	HyloM	PanP	PanTS	PanTT	PanTV	PongoA	PongoP	SymS
**Hool**	0	207.37	205.82	208.91	216.87	88.45	89.67	84.98	215.01	209.55	209.55	217	196.53	193.98	55.75
**GorBB**	207.37	0	4.63	29.32	86.66	247.12	248.34	243.65	84.8	79.34	79.34	86.79	172.52	169.97	214.42
**GorBG**	205.82	4.63	0	27.77	85.11	245.57	246.79	242.1	83.25	77.79	77.79	85.25	170.97	168.42	212.87
**GorGG**	208.91	29.32	27.77	0	88.2	248.66	249.88	245.19	86.34	80.88	80.88	88.33	174.06	171.51	215.96
**Homo**	216.87	86.66	85.11	88.2	0	256.62	257.84	253.15	79.16	73.7	73.7	81.15	182.02	179.47	223.92
**HyloA**	88.45	247.12	245.57	248.66	256.62	0	36.08	18.71	254.76	249.3	249.3	256.75	236.28	233.73	89.94
**HyloL**	89.67	248.34	246.79	249.88	257.84	36.08	0	32.61	255.98	205.52	250.52	257.97	237.5	234.95	91.16
**HyloM**	84.98	243.65	242.1	245.19	253.15	18.71	32.61	0	251.29	245.83	245.83	253.28	232.81	230.26	86.47
**PanP**	215.01	84.8	83.25	86.34	79.16	254.76	255.98	251.29	0	16.92	16.92	24.37	180.16	177.61	222.06
**PanTS**	209.55	79.34	77.79	80.88	73.7	249.3	205.52	245.83	16.92	0	0.73	12.05	174.7	172.15	216.6
**PanTT**	209.55	79.34	77.79	80.88	73.7	249.3	250.52	245.83	16.92	0.73	0	12.05	174.7	172.15	216.6
**PanTV**	217	86.79	85.25	88.33	81.15	256.75	257.97	253.28	24.37	12.05	12.05	0	182.15	179.6	224.05
**PongoA**	196.53	172.52	170.97	174.06	182.02	236.28	237.5	232.81	180.16	174.7	174.7	182.15	0	11.57	203.58
**PongoP**	193.98	169.97	168.42	171.51	179.47	233.73	234.95	230.26	177.61	172.15	172.15	179.6	11.57	0	201.03
**SymS**	55.75	214.42	212.87	215.96	223.92	89.94	91.16	86.47	222.06	216.6	216.6	224.05	203.58	201.03	0

*Taxon codes: Hool, *Hoolock hoolock*; GorBB, *Gorilla beringei beringei*; GorBG, *Gorilla beringei graueri*; GorGG, *Gorilla gorilla*; Homo, *Homo sapiens*; HyloL, *Hylobates lar*; HyloM, *Hylobates muelleri*; HyloA, *Hylobates agilis*; Pan TT; *Pan troglodytes troglodytes*; PanTS, *Pan troglodytes schweinfurthii*; PanTV, *Pan troglodytes verus*; PanP, *Pan paniscus*; PongoA, *Pongo abelii*; PongoP, *Pongo pygmaeus*; SymS, *Symphalangus syndactylus*.
