# Supplementary material for: Is the human chin a spandrel? Insights from an evolutionary analysis of ape craniomandibular form
Source: PLoS One. 2026 Jan 29;21(1):e0340278. doi: 10.1371/journal.pone.0340278 (PMC12854472; doi:10.1371/journal.pone.0340278)
Supplement: S6 Table — Node numbers and ancestor taxon codes match those used in S5 Table. (PDF) [file pone.0340278.s006.pdf]

S6 Table. Divergence times, generation times, and effective population sizes (with 90% confidence intervals) for each ancestral node. Node numbers and ancestor taxon codes match those used in S5 Table.

Node	Ancestor	Divergence times (mya) †	Divergence time ranges*	Generation times ‡	N_e §	Estimated N_e (90% CI)
1	PanTTPanTS	0.30		25	19,000	13,350 (7,306-19,401)
2	PanT	1.24		25	7,500	18,235 (11,592-24,966)
3	Pan	2.17	2.8 (1.93-3.72)	25	30,500	23,127 (15,833-30,472)
4	HomoPan	6.60	7.7 (5.70-9.45)	29	56,500	46,287 (36,034-56,697)
7	GorHomoPan	8.30	9.9 (7.60-12.11)	20	48,500	66,023 (53,248-79,045)
5	GorBBGorBG	0.40		19		14,534 (8,338-20,741)
6	Gor	1.80		19	21,000	24,164 (16,738-31,646)
9	PongoGorHomoPan	16.52	18.1 (15.41-20.98)	20	125,000	119,740 (100,102-139,873)
8	Pongo	1.31	2.6 (1.52-3.88)^	26	19,000	18,367 (11,682-25,082)
10	HyloAHyloM	1.86		15	*23,616*	27,989 (20,074-35,977)
11	Hylo	3.26		15	*37,051*	40,187 (30,714-49,791)
12	SymHylo	8.60		15		86,717 (71,298-102,479)
13	HoolSymHylo	8.93	7.4 (5.49-9.32)	15		89,592 (73,806-105,735)
14	HomAncestor	20.32	20.5 (17.64-23.52)	11		253,221 (216,527-291,022)

† mya = millions of years ago. See Schroeder & von Cramon-Taubadel 2017 for details on divergence estimates.
* For comparison, these means and confidence intervals of estimated divergence times are taken from recent whole-genome analyses of primates (Shao et al. [1])°.
^ while this range of estimates for divergence times falls above the mean used in this study, Prado-Martinez et al. [2] suggests a more recent divergence time of 0.97 mya and a recent study (Yoo et al. [3]) provides an estimate of 0.96 mya for the split between the two species of *Pongo*.
‡ Generation times are taken from Langergraber et al. [4] for hominids and from Chan et al. [5] for hylobatids.
§ Effective population sizes for splits taken from Prado-Martinez et al. [2]. These data were only available for some of the great ape nodes and were used to create a regression equation for the relationship between N_e and t (divergence time/generation time) in order to generate estimated N_e values (90% confidence intervals)

for all nodes. Available N_e estimates for lesser ape nodes from Chan et al. [5] are also shown (in italics) to illustrate that these estimates also fall well within the range of estimated N_e employed. See Schroeder & von Cramon-Taubadel [6] for further details.

°1. Shao Y, Zhou L, Li F, Zhao L, Zhang B-L, Shao F, et al. Phylogenomic analyses provide insights into primate evolution. Science. 2023;380(6648):913-24; 2.Prado-Martinez J, Sudmant PH, Kidd JM, Li H, Kelley JL, Lorente-Galdos B, et al. Great ape genetic diversity and population history. Nature. 2013;499:471-5; 3.Yoo D, Rhie A, Hebbar P, Antonacci F, Logsdon GA, Solar SJ, et al. Complete sequencing of ape genomes. Nature. 2025;641(8062):401-18. doi: 10.1038/s41586-025-08816-3. 4. Langergraber KE, Prüfer K, Rowney C, Boesch C, Crockford C, Fawcett K, et al. Generation times in wild chimpanzees and gorillas suggest earlier divergence times in great ape and human evolution. Proceedings of the National Academy of Sciences, USA. 2012;109(39):15716-21. 5.Chan Y-C, Roos C, Inoue-Murayama M, Inoue E, Shih C-C, Pei KJ-C, et al. Inferring the evolutionary histories of divergences in *Hylobates* and *Nomascus* gibbons through multilocus sequence data. BMC Evolutionary Biology. 2013;13:82. 6.Schroeder L, von Cramon-Taubadel N. The evolution of hominoid cranial diversity: A quantitative genetic approach. Evolution. 2017;71(11):2634-49.
